# Clinical, biochemical and molecular analysis of 13 Japanese patients with β-ureidopropionase deficiency demonstrates high prevalence of the c.977G > A (p.R326Q) mutation

**DOI:** 10.1007/s10545-014-9682-y

**Published:** 2014-02-14

**Authors:** Yoko Nakajima, Judith Meijer, Doreen Dobritzsch, Tetsuya Ito, Rutger Meinsma, Nico G. G. M. Abeling, Jeroen Roelofsen, Lida Zoetekouw, Yoriko Watanabe, Kyoko Tashiro, Tomoko Lee, Yasuhiro Takeshima, Hiroshi Mitsubuchi, Akira Yoneyama, Kazuhide Ohta, Kaoru Eto, Kayoko Saito, Tomiko Kuhara, André B. P. van Kuilenburg

**Affiliations:** 1 Department of Clinical Chemistry, Laboratory Genetic Metabolic Diseases, Academic Medical Center, 1105 AZ Amsterdam, Netherlands; 2 Department of Pediatrics and Neonatology, Nagoya City University Graduate School of Medical Sciences, 467-8601 Nagoya, Japan; 3 Department of Chemistry, Uppsala University, BMC, 75123 Uppsala, Sweden; 4 Department of Pediatrics and Child Health, Kurume University School of Medicine, 830-0011 Kurume, Japan; 5 Research Institute of Medical Mass Spectrometry, Kurume University School of Medicine, 830-0011 Kurume, Japan; 6 Department of Pediatrics, Kobe University Graduate School of Medicine, 650-0017 Kobe, Japan; 7 Department of Neonatology, Kumamoto University Hospital, 860-8556 Kumamoto, Japan; 8 National Rehabilitation Center for Disabled Children, 173-0037 Tokyo, Japan; 9 Department of Pediatrics, National Hospital Organization Kanazawa Medical Center, 920-8650 Kanazawa, Japan; 10 Department of Pediatrics, Tokyo Women’s Medical University, 162-8111 Tokyo, Japan; 11 Institute of Medical Genetics, Tokyo Women’s Medical University, 162-0054 Tokyo, Japan; 12 Japan Clinical Metabolomics Institute, 929-1174 Kahoku, Japan

## Abstract

β-ureidopropionase (βUP) deficiency is an autosomal recessive disease characterized by N-carbamyl-β-amino aciduria. To date, only 16 genetically confirmed patients with βUP deficiency have been reported. Here, we report on the clinical, biochemical and molecular findings of 13 Japanese βUP deficient patients. In this group of patients, three novel missense mutations (p.G31S, p.E271K, and p.I286T) and a recently described mutation (p.R326Q) were identified. The p.R326Q mutation was detected in all 13 patients with eight patients being homozygous for this mutation. Screening for the p.R326Q mutation in 110 Japanese individuals showed an allele frequency of 0.9 %. Transient expression of mutant βUP enzymes in HEK293 cells showed that the p.E271K and p.R326Q mutations cause profound decreases in activity (≤ 1.3 %). Conversely, βUP enzymes containing the p.G31S and p.I286T mutations possess residual activities of 50 and 70 %, respectively, suggesting we cannot exclude the presence of additional mutations in the non-coding region of the *UPB1* gene. Analysis of a human βUP homology model revealed that the effects of the mutations (p.G31S, p.E271K, and p.R326Q) on enzyme activity are most likely linked to improper oligomer assembly. Highly variable phenotypes ranging from neurological involvement (including convulsions and autism) to asymptomatic, were observed in diagnosed patients. High prevalence of p.R326Q in the normal Japanese population indicates that βUP deficiency is not as rare as generally considered and screening for βUP deficiency should be included in diagnosis of patients with unexplained neurological abnormalities.

**Electronic supplementary material:**

The online version of this article (doi:10.1007/s10545-014-9682-y) contains supplementary material, which is available to authorized users.

## Introduction

Pyrimidine nucleotides play an important role in various biological processes, including synthesis of RNA, DNA, phospholipids, uridine diphosphate glucose and glycogen. Intracellular pools of pyrimidines are produced de novo through salvage and catabolic pathways (Huang and Graves 2003; Traut 1994), and in humans, the pyrimidine bases uracil and thymine, are degraded via three enzymatic steps (Wasternack 1980). Dihydropyrimidine dehydrogenase (DPD, EC 1.3.1.2) is the initial and rate-limiting enzyme, catalyzing uracil and thymine reduction to 5,6-dihydrouracil and 5,6-dihydrothymine, respectively. The second enzyme, dihydropyrimidinase (DHP, EC 3.5.2.2), catalyzes the hydrolytic ring opening of the dihydropyrimidines. The third step, catalyzed by β-ureidopropionase (βUP) (EC 3.5.1.6), results in conversion of N-carbamyl-β-alanine and N-carbamyl-β-aminoisobutyric acid into β-alanine and β-aminoisobutyric acid, respectively, with concomitant production of ammonia and carbon dioxide.

Higher eukaryotic βUP belong to the nitrilase superfamily of enzymes (Pace and Brenner 2001). The closest known structural relative of human βUP is found in *Drosophila melanogaster* (DmβUP) (Lundgren et al 2008), sharing 63 % amino acid sequence identity. In solution, DmβUP exists as a mixture of oligomers but crystallizes as a homooctamer. It has a helical-turn like structure that is consecutively built up from dimeric units. This is in contrast to other members of the nitrilase superfamily that assemble their homotetrameric or homohexameric native states in a markedly different fashion, and is most likely because of an N-terminal ∼65 amino acid extension unique to βUPs.

βUP deficiency (MIM 606673) is an autosomal recessive disease caused by mutations in the βUP gene, *UPB1*. The *UPB1* gene maps to chromosome 22q11.2, and consists of ten exons spanning approximately 20 kb of genomic DNA (Vreken et al 1999). To date, only 16 genetically confirmed patients with βUP deficiency have been reported (van Kuilenburg et al 2012). The clinical phenotype of these patients is highly variable, but tends to center around neurological problems (van Kuilenburg et al 2012). However, in Japan, four asymptomatic individuals have been detected through newborn screening by gas chromatography-mass spectrometry (GC/MS), and the prevalence of βUP deficiency in Japan has been estimated to be one in 6000 (Kuhara et al 2009). Thus, the clinical presentation and biochemical and genetic spectrum of patients with βUP deficiency are still largely unknown.

In this study, we report genetic and biochemical analysis, and clinical follow-up findings, of 13 Japanese patients (including seven newly identified individuals) with βUP deficiency. Functional and structural consequences of the mutations at the protein level were analysed using a eukaryotic expression system and a homology model generated based on the crystal structure of recombinant DmβUP.

## Materials and methods

### Patients

Patients 1, 2 and 3, who presented with neurological abnormalities during early childhood were detected through a high-risk urine screening for general metabolic disorders performed at Kanazawa Medical University (Ohse et al 2002). In general patients are tested for metabolic disorders if patients presented with developmental delay, hyperammonemia, metabolic acidosis and neurological manifestations such as convulsions, autism and related disorders. Patients 4–7 and 8–13 were from two different areas in Japan, detected in a pilot study screening for inborn errors of metabolism by GC/MS in newborn urine samples, and conducted at Kanazawa Medical University (Kuhara et al 2009) (patients 4–7) and Kurume University (patients 8–13). After informed consent was obtained from their parents, urine and blood samples from all patients were sent to the Laboratory for Genetic Metabolic Diseases in Amsterdam, the Netherlands for further analysis.

### Quantitative pyrimidine analysis

On the basis of a gross elevation of N-carbamyl-ß-alanine and N-carbamyl-ß-aminoisobutyric acid in urine screening for inborn errors of metabolism by GCMS (Ohse et al 2002), ß-ureidopropionase deficiency was suspected. Subsequently, quantitation of relevant pyrimidines and its metabolites was performed by HPLC tandem mass spectrometry. Concentrations of uracil, thymine, dihydrouracil, dihydrothymine, N-carbamyl-β-alanine and N-carbamyl-β-aminoisobutyric acid, in urine-soaked filter paper strips, were determined using reversed-phase HPLC-tandem mass spectrometry (HPLC-MS/MS) (van Kuilenburg et al 2004c; van Lenthe et al 2000).

### PCR amplification of *UPB1* coding exons

DNA was isolated from whole blood or blood spots using the QIAamp DNA Micro kit (QIAGEN). Exons 1-10 and flanking intronic regions of *UPB1*, were amplified using previously described primer sets (van Kuilenburg et al 2004a). *UPB1* sequence from patients was compared to controls and the reference *UPB1* sequence (Ref Seq NM_016327.2).

### Cloning and site-directed mutagenesis

An expression plasmid containing wild-type human βUP cDNA (pSE420-βUP) was constructed by subcloning the complete coding region of human *UPB1* into the *Nco*I-*Nae*I site of the pSE420 vector (Vreken et al 1999). The *UPB1* coding sequence was then re-cloned into the *BamH*I-*Kpn*I site of the pcDNA3.1Zeo vector, which includes coding sequence for a cleavable C-terminal 6-His-fusion tag. To introduce mutations, the pcDNA3.1Zeo plasmid containing wild-type *UPB1* was subjected to site-directed mutagenesis. Compatible primers (Table 1S, Supplementary data) were designed for use with the QuikChange™ Site-Directed Mutagenesis Kit (Life technologies). PCR-mediated site-directed mutagenesis was performed according to the manufacturer’s recommended protocol. All resulting plasmids were sequenced to confirm introduction of single nucleotide changes.

### Cell culture and transient transfection

HEK293 cells were cultured in Dulbecco’s modified eagle’s medium with 4.5 g/L glucose, 25 mM Hepes and 584 mg/L L-glutamine (Lonza), supplemented with 10 % fetal bovine serum, 100 U/ml penicillin, 100 mg/ml streptomycin and 250 μg/ml fungizone at 37 °C in a humidified 5 % CO_2_ incubator. For transient transfections, cell cultures were set up in six-well plates 24 h prior to transfection. HEK293 cells were transfected with pcDNA3.1Zeo-βUP (wild-type or variants) using X-treme GENE HP DNA Transfection reagent (Roche). Two days after transfection, cells were harvested and washed with PBS. After centrifugation at 1000 × g for 5 min at 4 °C, cell pellets were immediately frozen in liquid nitrogen and stored at−80 °C until use. All transfections were performed in at least triplicate. Parental vector (pcDNA3.1Zeo) without insert was transfected as negative control.

### βUP enzyme activity assay

Cell pellets were resuspended in 300 μl isolation buffer (35 mM potassium phosphate, pH 7.4 and 2.5 mM MgCl_2_) and lysed by sonication on ice. Crude lysates were centrifuged at ≥ 11,000 rpm for 20 min at 4 °C, and then supernatant protein concentrations and βUP enzyme activity of the expressed protein directly quantified. βUP activity was determined at 37 °C in a standard assay mixture containing cell supernatant, 200 mM Mops (pH 7.4), 1 mM dithiothreitol and 500 μM [^14^C]-N-carbamyl-β-alanine, as described previously (Van Kuilenburg et al 1999).

### Western blot analysis

Cell supernatants containing 5 μg protein were fractionated on NuPAGE® 4–12 % Bis-Tris Mini Gels (Life technologies) and transferred to nitrocellulose membranes. Membranes were blocked using Odyssey blocking buffer (LI-COR). Subsequently, blots were incubated for one hour with a 1:1000 dilution of rabbit anti-UPB1 (Anti-UPB1 AV42467-100UG, Sigma-Aldrich) and 1:5000 dilution of mouse anti-alpha-tubulin antibodies in blocking buffer (50 % Odyssey blocking buffer, 50 % PBS and 0.1 % Tween). Membranes were washed three times and then incubated for one hour with a 1:10,000 dilution of IRDye800 conjugated goat anti-rabbit and IRDye680 conjugated donkey anti-mouse (both LI-COR) secondary antibodies, in the same blocking buffer as used for primary antibodies, with 0.01 % SDS. Blots were scanned and band intensities analysed using the LI-COR Odyssey infrared imaging system.

### Native gel electrophoresis

Blue native polyacrylamide gel electrophoresis was performed using 4–16 % NativePAGE™ Novex® Bis-Tris Gels (Life technologies). Supernatant samples were prepared in sample buffer (50 mM Bis Tris, 6 N HCL, 50 mM NaCl, 10 % glycerol and 0.001 % Ponceau S, pH 7.2), and 5 μg protein loaded. Electrophoresis was performed at 100 V for 2 h at room temperature. Gels were transferred onto PVDF membranes and immunoblotting performed, as described above. NativeMark™ Unstained Protein Standard (Life technologies) was used as a molecular weight marker, and visualized with Ponceau S staining after western transfer.

### Crystal structure analysis

**Fig. 1:**
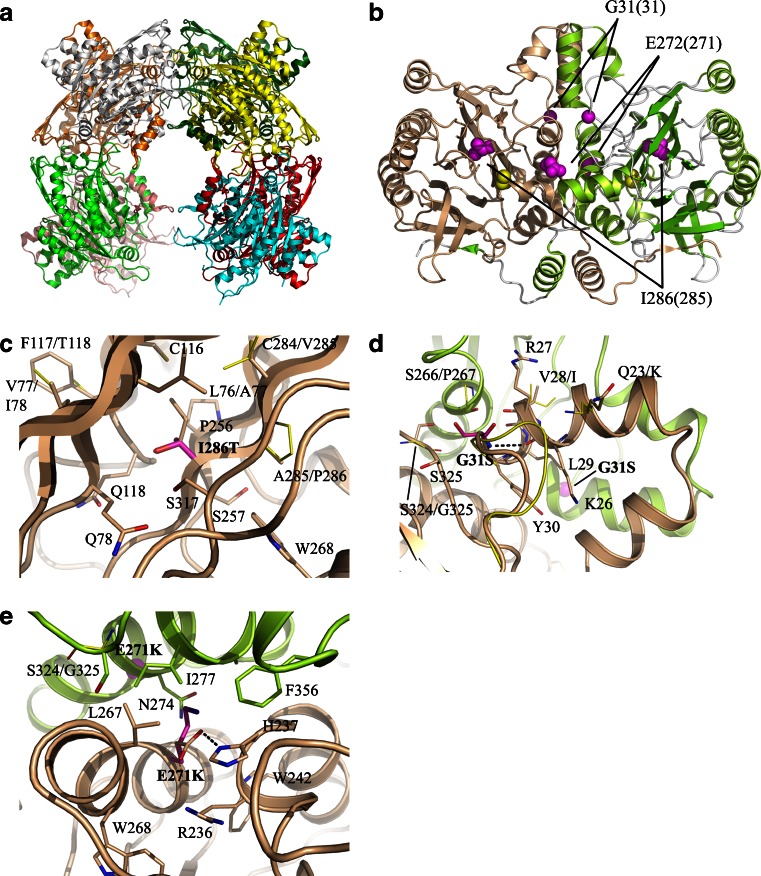
DmβUP crystal structure and mutation site environment in the homology model of human βUP. (**a**) Schematic view of homooctameric DmβUP with each subunit coloured differently. (**b**) Schematic view of a dimeric unit of DmβUP. For one of the subunits, β-strands are depicted in green, helices in yellow-green and loops in white, with the other subunit coloured salmon. Mutation sites are highlighted by space-filling models of the respective amino acid side chains in magenta. Labels first list the corresponding site in DmβUP. Location of the active site is indicated by space-filling models of the active site cysteine (C233 in human βUP, C234 in DmβUP) in yellow. (**c**-**e**) Enlarged views of I286T, G31S and E271K mutation sites. The homology model of human βUP is shown with different colours (salmon and green) for two separate subunits. Additional subunits were omitted as none of the mutations occur near putative interfaces. Stick models of side chains introduced by the mutations are shown in magenta, in preferred conformations causing the least clashes. Native side chains and residues surrounding the site are depicted with carbon atoms in the same colour as the subunit to which they belong. DmβUP side chains are shown with yellow carbon atoms when not conserved in human βUP. Labels indicate human βUP residues followed by corresponding DmβUP residues (if shown), with numbering for the latter only when it differs. Hydrogen bonds are indicated by dotted black lines. In (d), the loop directly following the G31S site is extended by one amino acid in DmβUP (shown in yellow). In (d) and (e), corresponding mutation sites in the second subunit of the dimer are marked by a magenta sphere in the background

A homology model of human βUP was generated using the SWISSMODEL server, based on the crystal structure of *Drosophila melanogaster* βUP (DmβUP) (PDB-ID:2vhi and 2vhh) (Lundgren et al 2008). WinCoot (Emsley et al 2010) was used for structural analysis and manual introduction of amino acid exchanges resulting from *UPB1* mutations. Energetically preferred side chain conformations causing the least steric clashes and optimal interactions with surrounding residues were chosen. Figure 1 was generated using PyMol (DeLano 2002).

### Genotyping of c.977G > A by PCR-based restriction fragment length polymorphism (PCR-RFLP) analysis

A 333 bp fragment of genomic DNA was amplified using a primer set used for mutation analysis of exon 9 of the *UPB1* gene. The amplified product was digested using the restriction enzyme *Msp*I, for 2 h at 37 °C. Transition of G to A at c.977 abolishes a restriction site in the mutant allele. Digested products were separated on 2.8 % agarose gels stained with ethidium bromide. Genotypes were identified as wild-type (GG) (three bands of 192, 153 and 24 bp), heterozygous mutant (GA) (bands of 216, 192, 153 and 24 bp), or homozygous mutant (AA) (bands of 216 and 153 bp).

In addition, the frequency of the c.977G > A mutation was assessed in the exome variant server (EVS) of the National Heart, Lung, and Blood Institute GO Exome Sequencing Project (Seattle, WA, USA; URL: evs.gs.washington.edu/EVS/) with the corresponding nucleotide positions being analysed in >8.000 European and >4.000 African American alleles, and in the Database of Single Nucleotide Polymorphisms (dbSNP; Bethesda: National Center for Biotechnology Information, National Library of Medicine [dbSNP Build ID: 137]; available from: http://www.ncbi.nlm.nih.gov/SNP/).

## Results

### Clinical evaluation

All patients were born to healthy non-consanguineous Japanese parents. Patient 1 was a girl born at full term following an uneventful delivery. At the age of two months, she was irritable and had occasional jerky eye movements, with impairment of visual contact noticed. A week later she presented with infantile spasms, and head-nodding three to four times a day. Cortical dysplasia was suspected from head MRI, and EEGs showed hypsarrhythmia. She was diagnosed with West syndrome. Biochemical investigation of urine obtained during her first admission revealed significantly increased levels of N-carbamyl-β-alanine and N-carbamyl-β-aminoisobutyric acid (Kuhara et al 2009). The infantile spasms and abnormal EEGs did not respond to zonisamide, therefore adrenocorticotropic hormone (ACTH) therapy was started at three months of age, and subsequently, the seizures disappeared and EEG abnormalities subsided. At the age of 5 years, she had partial seizures occasionally but her development was within the normal range.

Patient 2 was a 3-year old boy who presented with autism and mild mental retardation. At the age of 18 months, his parents noticed social communication impairments, including eye-to-eye gazing, facial expression, and language understanding. At the age of 3.5 years, he was diagnosed with autism. When he was 8 years old, he developed a sleep disorder and melatonin treatment was started. The Wechsler Intelligence Score for Children test, performed at 11 years of age, revealed a full scale IQ of 71, verbal IQ of 66, and performance IQ of 83. He now attends a special-needs school.

Patient 3 was a 12-month old boy who presented with mild hypotonia and motor developmental delay. The family history found a 15-year old male cousin from the paternal side suffers from epilepsy and autism related disorder. At the follow-up age of 2.8 years, his motor development caught up to the normal range but he has developed mild mental retardation, particularly in speech development.

Patients 4–7 were detected through neonatal screening performed from 1996 to 2009 in Kanazawa (Kuhara et al 2009). Since screening, they have been followed regularly by an attending paediatrician. Patient 4 is a boy who developed neurological symptoms during the follow-up period. At the age of 12 months, he had an attack of sudden-onset consciousness impairment, a blank stare, and unresponsiveness which lasted for 2–3 min. A year after this episode, he developed febrile seizures that occurred four times. One of the attacks lasted over 30 min and was not easily controlled. The laboratory investigation at admission showed no abnormalities. His fever abated the following day and he regained consciousness. Further investigations by head MRI and EEG were normal. He has had no seizures since 3 years of age, owing to the use of diazepam suppositories during fevers. At 5 years of age, he has normal growth and development.

Patients 5 and 6 are twin sisters, born at 36 weeks gestation with birth weights of 2042 g and 2378 g, respectively. During a follow-up period of 5 years, patient 6 showed no physical examination abnormalities. Patient 5 has a height within the 3–10 percentile range and has developed hypermetropia. Patient 7 is a 10-year-old boy with an unremarkable history and no clinical manifestations during follow-up. Patients 8–13 were detected from newborn screening performed in Kurume during a period from February 2010 to January 2012. None of them showed abnormal developmental milestones at follow-up evaluation.

### Pyrimidine bases and degradation metabolites in urine

**Fig. 2:**
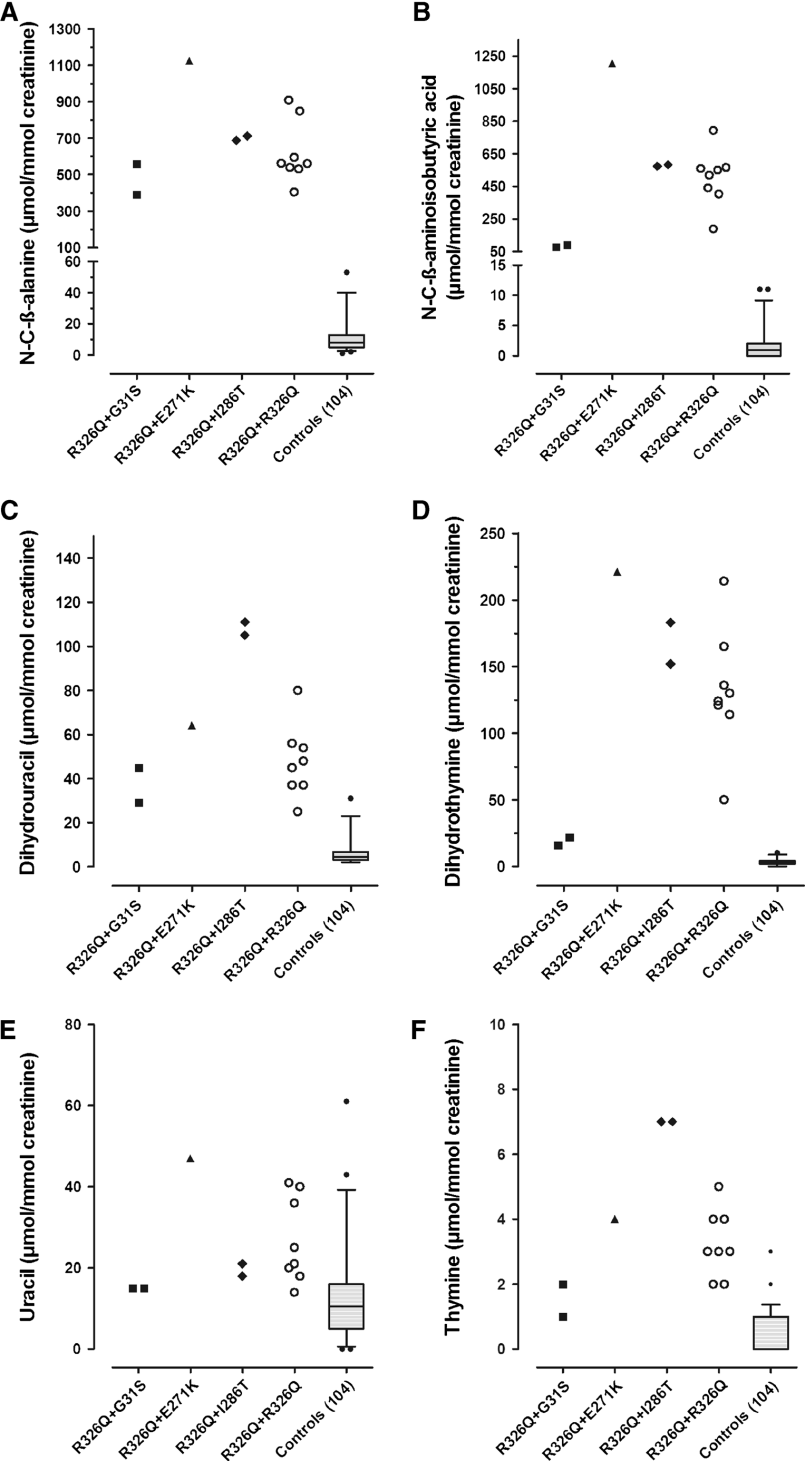
Pyrimidine and metabolite concentrations in urine of βUP deficient patients and controls. Patients are classified in terms of their genotype. (**a**) N-carbamyl-β-alanine, (**b**) N-carbamyl-β-aminoisobutyric acid, (**c**) dihydrouracil, (**d**) dihydrothymine, (**e**) uracil, and (**f**) thymine. In controls, the top, bottom and line through the middle of a box, correspond to the 75th, 25th and 50th percentiles, respectively. Whiskers on the bottom extend from the 2.5th percentile, and on the top, the 97.5th percentile

Urinary quantitative analysis of relevant pyrimidines and metabolites was performed by HPLC-MS/MS. Urinary concentrations of the 13 patients are shown as subdivided genotype groups (Fig. 2). All urine samples from the patients showed strongly elevated levels of N-carbamyl-β-alanine and N-carbamyl-β-aminoisobutyric acid, and moderately elevated levels of dihydrouracil and dihydrothymine. Mean concentrations of uracil (25.5 ± 11.4 μmol/mmol creatinine), thymine (3.6 ± 1.9 μmol/mmol creatinine), dihydrouracil (57 ± 27 μmol/mmol creatinine), and dihydrothymine (127 ± 65 μmol/mmol creatinine), were 2-, 7-, 9-, and 41–fold, respectively, higher compared with mean concentrations observed in controls. Additionally, mean concentrations of N-carbamyl-β-alanine (648 ± 208 μmol/mmol creatinine) and N-carbamyl-β-aminoisobutyric acid (504 ± 297 μmol/mmol creatinine) were 59-and 276-fold, respectively, higher compared to mean concentrations observed in controls. The observed N-carbamyl-β-amino aciduria in these patients strongly suggests βUP deficiency.

### Mutation analysis of *UPB1*

**Fig. 3:**
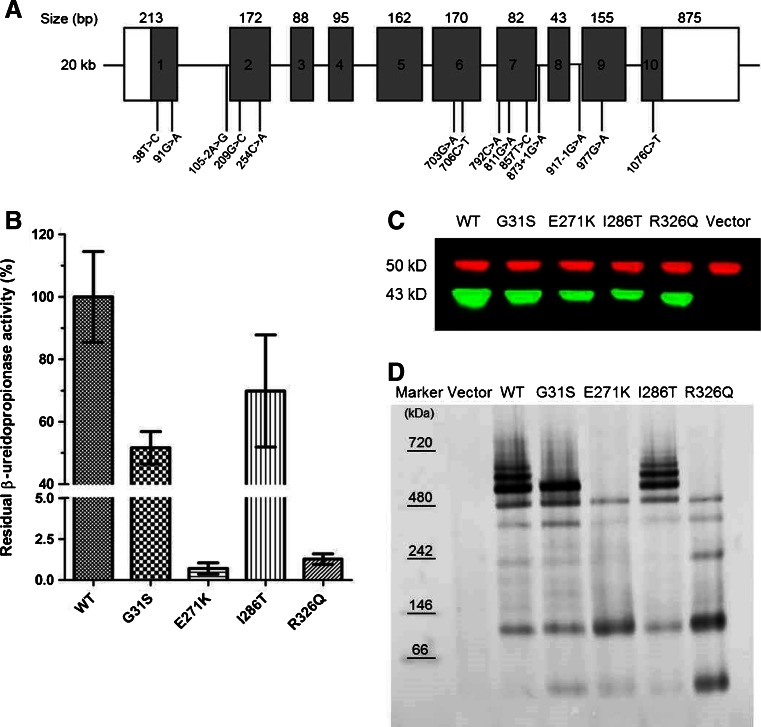
(**a**) Schematic representation of genomic organization of the *UPB1* gene. *UPB1* consists of ten exons encoding an open reading frame of 1152 bp (depicted in grey). The mutations identified to date in βUP deficient patients are indicated, with numbers corresponding to cDNA positions. (**b**) Expression of βUP mutants in HEK293 cells. Residual enzymatic activity of βUP mutants are expressed as percentages of wild-type βUP activity. For each construct, columns show mean values and standard deviations derived from at least three transfections. (**c**) Western blot analysis of HEK293 cells expressing wild-type and mutant βUP. Total cell protein (5 μg) was resolved by SDS-PAGE followed by immunoblotting against βUP and alpha-tubulin. (**d**) Native polyacrylamide gel electrophoresis of HEK293 cells expressing βUP protein (wild-type and mutants). Cell supernatants (5 μg) were subjected to 4–16 % blue native page, followed by western blot analysis using polyclonal anti-βUP antibody

Sequencing exons 1–10 (including flanking intronic regions) of *UPB1* in the 13 patients, identified one previously described missense (c.977G > A) and three novel missense (c.91G > A, c.811G > A, and c.857 T > C) mutations (Table 1). This table also includes 15 patients (from 12 families) with βUP deficiency, and previously published mutations (Assmann et al 1998; Assmann et al 2006; van Kuilenburg et al 2012; Yaplito-Lee et al 2008). All 13 Japanese patients were carriers of the c.977G > A (p.R326Q) mutation, with eight patients being homozygous for this mutation (Table 1). Distribution of the *UPB1* mutations within the exons is shown (Fig. 3a). All previously identified mutations and the three novel missense mutations were located in exons 1, 2, and 6–10.

**Table 1:** Genetic and phenotypic findings of patients with β-ureidopropionase deficiency

Patient No.	Origin	Consanguinity	Sex	Age at diagnosis (years)	Age at follow-up (years)	Symptom	Genotype	Effect	Location	Reference
1	Japan	-	F	0.2	5.0	Seizures (West syndrome)	c.[977G > A] + [977G > A]	p.[R326Q] + [R326Q]	Ex 9	van Kuilenburg, et al 2012
2	Japan	-	M	3.5	16.0	MR, Autism	c.[977G > A] + [977G > A]	p.[R326Q] + [R326Q]	Ex 9	Present study
3	Japan	-	M	1.0	2.8	Motor retardation MR	c.[811G > A] + [977G > A]	p.[E271K] + [R326Q]	Ex 7, Ex 9	Present study
	Turkey^a)^	+	M	0.8	NA	Seizures	c.[1076C > T] + [1076C > T]	p.[T359M] + [T359M]	Ex 10	van Kuilenburg, et al 2012
	Egypt^b)^	+	F	Birth	NA	Seizures, MC	c.[105-2A > G] + [105-2A > G]	splicing	Int 1	van Kuilenburg, et al 2012
	Egypt^b)^	+	M	Birth	NA	Seizures, MC	No DNA available			van Kuilenburg, et al 2012
	Egypt	+	M	0.8	NA	Seizures, MR, hypotonia	c.[38 T > C] + [38 T > C]	p.[L31S] + [L31S]	Ex 1	van Kuilenburg, et al 2012
	Pakistan	+	F	2.0	NA	MC, MR, hypotonia, Autism	c.[792C > A] + [873 + 1G > A]	p.[S264R] + splicing	Ex7, Int 7	van Kuilenburg, et al 2012
	China	−	M	1.1	NA	MC, MR	c.[977G > A] + [977G > A]	p.[R326Q] + [R326Q]	Ex 9	van Kuilenburg, et al 2012
	Germany	−	F	0.9	NA	Seizures, hypotonia	c.[703 > A] + [917-1G > A]	p.[G235R] + splicing	Ex 6, Int 8	van Kuilenburg, et al 2012
	China	−	M	3.0	NA	MR	c.[706C > T] + [792C> A]	p.[R236W] + [S264R]	Ex 6, Ex 7	van Kuilenburg, et al 2012
	Turkey	+	F	5.3	NA	MR, hypotonia	c.[105-2A > G] + [917-1G > A]	splicing	Int 1, Int 8	Assmann et al 1998; van Kuilenburg 2004
	Turkey	+	F	3.0	NA	Seizures, MR	c.[105-2A > G] + [105-2A > G]	splicing	Int 1	van Kuilenburg 2004
	Germany	−	M	0.9	NA	Seizures, MC, MR, hypotonia	c.[917-1G > A] + [917-1G > A]	splicing	Int 8	Assmann et al 2006; van Kuilenburg 2004
	African	−	F	1.0	NA	Seizures	c.[254C > A] + [254C > A]	p.[A85E] + [A85E]	Ex 2	van Kuilenburg 2004
	Australia	−	M	1.0	NA	Urogenital and colorectal system anomalies	c.[209G > C] + [105-2A > G]	p.[R70P] + splicing	Ex 2 Int 1	Yaplito-Lee et al 2008
	Turkey^a)^		F	30, CT	NA	AS	c.[1076C > T] + [1076C > T]	p.[T359M] + [T359M]	Ex 10	van Kuilenburg, et al 2012
	Egypt^b)^		M	27, CT	NA	AS	c.[105-2A > G] + [105-2A > G]	splicing	Int 1	van Kuilenburg, et al 2012
4	Japan	−	M	NS	5.3	Absence seizure, febrile seizure	c.[977G > A] + [977G > A]	p.[R326Q] + [R326Q]	Ex 9	Present study
5*	Japan	−	F	NS	5.5	AS	c.[857 T > C] + [977G > A]	p.[I286T] + [R326Q]	Ex7, Ex9	Present study
6*	Japan	−	F	NS	5.5	AS, hypermetropia	c.[857 T > C] + [977G > A]	p.[I286T] + [R326Q]	Ex7, Ex9	Present study
7	Japan	−	M	NS	10.5	AS	c.[977G > A] + [977G > A]	p.[R326Q] + [R326Q]	Ex 9	Present study
8	Japan	−	M	NS	2.5	AS	c.[977G > A] + [977G > A]	p.[R326Q] + [R326Q]	Ex 9	Present study
9	Japan	−	F	NS	2.9	AS	c.[977G > A] + [977G > A]	p.[R326Q] + [R326Q]	Ex 9	Present study
10	Japan	−	M	NS	1.3	AS	c.[91G > A] + [977G > A]	p.[G31S] + [R326Q]	Ex1, Ex 9	Present study
11	Japan	−	F	NS	2.3	AS	c.[977G > A] + [977G > A]	p.[R326Q] + [R326Q]	Ex 9	Present study
12	Japan	−	F	NS	1.7	AS	c.[977G > A] + [977G > A]	p.[R326Q] + [R326Q]	Ex 9	Present study
13	Japan	−	F	NS	1.1	AS	c.[91G > A] + [977G > A]	p.[G31S] + [R326Q]	Ex1, Ex 9	Present study

NA = not available, NS = neonatal screening, CT = carrier testing, AS = asymptomatic, MR = mental retardation, MC = microcephaly,

* Patient 5 and 6 are twin siblings. ^a)^ indicates same family members (child and mother). ^b)^ indicates same family members (two siblings and father)

Biochemical data of patient 1, 2, 4, 5, 6 and 7 were previously reported (Kuhara et al 2009)

### Functional analysis and expression of mutant βUP protein

Recombinant wild-type and four βUP proteins containing the mutations, p.G31S, p.E271K, p.I286T, and p.R326Q, were expressed in HEK293 cells. No endogenous βUP activity (< 0.7 nmol/mg/h) was detected in HEK293 cells. Activity of wild-type βUP protein was 1976 ± 416 nmol/mg/h (n = 9). Recombinant βUP enzymes carrying mutations p.E271K and p.R326Q, exhibited 0.7 and 1.3 %, respectively, of wild-type activity, whereas βUP enzymes containing mutations p.G31S and p.I286T, possessed residual activities of 51.6 and 69.8 %, respectively (Fig. 3b).

Immunoblot analysis of expression levels of βUP mutant proteins in soluble extracts from transfected HEK293 cells, showed mutant proteins were expressed in comparable amounts as wild-type protein (Fig. 3c).

Western blotting of blue native gels detected various high molecular weight oligomeric forms of wild-type βUP, comparable or identical to that observed for βUP enzymes containing p.G31S and p.I286T mutations. In contrast, no distinct sharp bands of high molecular weight were observed for βUP enzymes containing p.E271K and p.R326Q mutations, although bands of lower molecular weights were present, corresponding to lower oligomeric states (from monomers to octamers) of βUP (Fig. 3d). These lower molecular weight species were also observed in extracts from wild-type, p.G31S and p.I286T expressing cells, albeit with evidently lower intensity. This indicates that p.E271K and p.R326Q proteins have a dramatically reduced ability to form larger oligomers, and thus, their potential equilibrium of oligomerization status was shifted towards lower molecular weight species.

### Population study of βUP deficiency and the p.R326Q mutation

Three patients with βUP deficiency were identified out of 4500 patients of a high-risk screening group demonstrating that the prevalence of βUP deficiency in a high-risk screening group is one in 1500. PCR-RFLP analysis of the c.977G > A (p.R326Q) mutation was performed in 110 Japanese healthy controls. We identified two individuals heterozygous for the p.R326Q mutation, and no homozygous individuals, resulting in a frequency of heterozygotes in the Japanese population of 1.8 % (an allele frequency of 0.91 %). Analysis of publically available databases showed that the c.977G > A mutation was not detected in >8.000 European and >4.000 African American alleles whereas the mutation was detected with an allele frequency of 2.6 % in 286 individuals of East Asian ancestry.

### Analysis of the structural effects of *UPB1* mutations by homology modelling

A homology model of human βUP was generated to predict the effect of the *UPB1* mutations on enzyme structure. High sequence similarity between homologous enzymes at mutation sites (with G31 and E271 being strictly conserved in deposited βUP sequences, and I286 replaced by leucine in rodent enzymes only) suggests the mutations occur within structurally conserved regions.

I286 is located in the subunit core, approximately at 10 Å distance from the active site (Fig. 1b). It is surrounded by hydrophobic and polar residues (Fig. 1c). The increase in side chain polarity upon mutation to threonine may therefore be tolerated, and structural adjustments limited to orientation of the hydroxyl group towards polar neighbours and re-optimization of side chain packing, with minor effects on active site geometry and enzymatic activity. This correlates well with the measured residual activity, and unaltered behaviour of the mutant protein in native gel analysis.

G31 is located directly downstream of a helix involved in dimer interface formation in DmβUP (Fig. 1d) (Lundgren et al 2008). Mutation to serine requires structural rearrangement as the close proximity of S325 leaves little space for a side chain. Taking into account that several previously identified deleterious mutations also cluster at oligomerization surfaces (van Kuilenburg et al 2012), the significant loss in enzyme activity is most likely linked to disturbance of subunit interactions. Interestingly, native gel electrophoresis showed that only formation of larger assemblies (those exceeding the size of octamers) is hampered.

In DmβUP, the glutamate corresponding to E271 interacts with two basic residues that are both conserved in the human enzyme (H237, R236) (Fig. 1e). For the E271K mutant, clashes of lysine 271 with nearby residues, and placement of its positively charged head group in a hydrophobic (L267, I277 and F356) or similarly charged environment (H237 and R236), will have severely destabilizing effects, further amplified by the proximity of corresponding mutation sites of two subunits at the dimer interface (Fig. 1e). Structural changes to accommodate the altered side chain are likely to affect oligomerization, as confirmed by native gel electrophoresis (Fig. 3d). As the helix carrying E271 is placed beside the one harbouring C233, any shifts in its position may also directly influence active site geometry and cause the observed dramatic loss of enzymatic activity.

## Discussion

βUP deficiency is described as exhibiting variable phenotypic presentation, ranging from early infantile onset with severe neurological involvement, to mild developmental delay and learning disabilities, to asymptomatic individuals (van Kuilenburg et al 2012). Despite large variation in clinical presentation, the majority of previously identified patients (85 %) present with MRI abnormalities (van Kuilenburg et al 2012). In the present study, only three of the 13 patients had neurological problems during infancy, and underwent biochemical urine analysis as part of diagnostic examinations. Ten of the patients were identified through newborn screening programs. Follow-up clinical investigation revealed that one of these patients had suffered from an episode of unconsciousness, and later developed febrile seizures. To date, the nine other individuals have remained asymptomatic. As the follow-up period of some patients (specifically, patients 8–13) is relatively short, being only one to two years, it is conceivable that more patients may present with a clinical phenotype in due course.

The clinical phenotype of patients with DHP and DPD deficiencies, the other two biochemical defects occurring within the pyrimidine degradation pathway, is highly variable, ranging from severely (neurologically) affected to symptomless. The underlying pathogenesis of these variable clinical manifestations of pyrimidine degradation disorders remains, as yet, unknown. However, similarities between clinical phenotypes of patients with βUP deficiency, and DHP and DPD deficiencies, suggests loss of physiological function of the absent pathway metabolites, rather than toxicity of the accumulated metabolite, is the underlying cause. In this respect, patients with pyrimidine degradation defects show normal to slightly decreased β-alanine levels in plasma and normal levels in CSF, whereas β-aminoisobutyric acid concentrations are strongly reduced in plasma and CSF (van Kuilenburg et al 2004a, b, 2008). β-Aminoisobutyric acid is not only a partial agonist of the glycine receptor (Schmieden and Betz 1995), but has also been demonstrated to enhance leptin secretion in adipose cells (Begriche et al 2010). Leptin and its receptors are widespread within the central nervous system, and leptin has been shown to exert a neuroprotective effect in damaged brain regions (Signore et al 2008). Therefore, altered β-aminoisobutyric acid homeostasis in patients with pyrimidine degradation defects may contribute to neurological abnormalities. Treatment of a ßUP-deficient patient with ß-alanine for over 1.5 years did not result in a clinical improvement (Assmann et al 2006). So far, the clinical effect of ß-aminoisobutyric acid supplementation has not been investigated in ßUP-deficient patients. Considering the pivotal role of βUP in β-alanine and β-aminoisobutyric acid synthesis, we suspect it is unlikely that βUP deficiency is not related at all to the neurological presentation observed in some of our patients. However, our observation that patients with βUP deficiency can present without any clinical abnormalities suggests additional factors are involved in the clinical outcome. These additional factors likely include alterations in other genes and/or environmental factors.

Until now, eight missense and three splice site mutations in *UPB1* have been identified in patients with βUP deficiency (van Kuilenburg et al 2012). In this study, we identified three novel missense mutations and one recently reported mutation, p.R326Q, in 13 patients from 12 unrelated Japanese families. It is noteworthy that homozygosity of the p.R326Q mutation is observed in 62 % (8/13) of Japanese patients, and all 13 patients carried this mutation on one or both alleles, resulting in an allele frequency of 81 % (21/26) in Japanese patients with βUP deficiency. Based on the fact that 1.8 % of the Japanese population is heterozygous for the p.R326Q mutation, we estimate that one individual per 12,500 will be homozygous for the p.R326Q mutation. Thus, compared with other frequently occurring inborn errors of metabolism, such as phenylketonuria (1:70,000 in Japan), the expected prevalence of βUP deficiency is not as rare as generally considered.

The fact that none of the patients identified through newborn screening had lasting neurological problems, in combination with the high estimated prevalence in Japan and variable features in diagnosed patients, may indicate that the disease has low penetrance and is a risk factor. However, it may be too early to conclude that penetrance is low as we cannot exclude the possibility that these patients will develop symptoms later on in life. In addition, the prevalence of ß-ureidopropionase deficiency in a high-risk group is four-times higher (1:1500) than that observed in a control population (1:6000) (Kuhara et al 2009). This　observation suggests that ß-ureidopropionase deficiency might be involved in the onset of a clinical phenotype.

Expression of mutant βUPs showed that p.E271K and p.R326Q mutants exhibit significantly decreased residual activity, whereas p.G31S and p.I286T mutants have more than 50 % residual activity of wild type. This result is to some extent in agreement with the finding that the two patients heterozygous for p.G31S present with relatively low urinary N-carbamyl-β-aminoisobutyric acid concentrations, although this was not apparent in two siblings heterozygous for p.I286T (Fig. 2). In combination with the blue native gel analysis, there is no convincing evidence that the p.G31S or p.I286T mutations are pathogenic. Since no DNA of the parents was available, carriership analysis of the mutations could not be performed. Therefore, it is conceivable that c.91G > A (p.G31S) and c.857 T > C (p.I286T) are in *cis* with c.977G > A (p.R326Q). Thus, we cannot exclude the possibility that additional mutations may be present within non-coding regions of *UPB1* in patients carrying p.G31S or p.I286T mutations.

Recently, heterologous expression of p.R326Q mutant βUP in *E.coli* was shown to result in mutant enzyme with no residual activity (van Kuilenburg et al 2012). Similarly, in the present study, expression of p.R326Q mutant enzyme in HEK293 cells caused a dramatic decrease in residual activity. The wide-ranging urinary levels of N-carbamyl-β-alanine and N-carbamyl-β-aminoisobutyric acid observed in patients homozygous for the p.R326Q mutation (Fig. 2), suggest there is no clear correlation between urinary biochemical phenotype and genotype in this group of patients. In addition, identification of the p.R326Q mutation in both neurologically affected patients and unaffected individuals indicates the severity of βUP deficiency is not determined exclusively by *UPB1* mutant alleles alone, and other (epi)genetic factors modulate the effect of the final functional enzyme (Dipple and McCabe 2000; Sriram et al 2005).

Analysis of the homology model of human βUP, revealed that none of the mutation sites are found in or very near the active site. Instead, the two exchanges most deleterious to enzymatic activity occur at subunit surfaces that are buried upon dimerization. As a similar observation was made for previously reported point mutations of the human *UPB1* gene, it appears that proper subunit association of dimers or larger oligomers is required for full functionality of the encoded enzyme. This can most simply be explained in terms of enzyme stability. Alternatively, human βUP activity may be affected by ligand-induced changes in the oligomerization state, as described for the rat liver enzyme and bacterial homologues (Matthews et al 1992; Thuku et al 2007; Stevenson et al 1992; Nagasawa et al 2000). However, such potential regulatory properties of human βUP remain to be further investigated.

The pyrimidine degradation pathway is also responsible for degradation of the chemotherapeutic drug, 5-fluorouracil (5-FU). It is well known that patients with either complete or partial DPD deficiency can show severe toxicity after 5-FU administration (van Kuilenburg 2004; van Kuilenburg et al 2000). Furthermore, it has been demonstrated that patients with partial DHP deficiency are prone to develop severe 5-FU toxicity (van Kuilenburg et al 2003), and heterozygous mutations in the *UPB1* gene may impair uracil catabolism (Fidlerova et al 2012; Thomas et al 2008). Therefore, risk of developing 5-FU toxicity is not limited to DPD deficiency, and patients with βUP deficiency may also be at risk of developing severe 5-FU toxicity.

Our study shows that even though the clinical manifestation of βUP deficient patients varies considerably from symptomless to severely neurologically affected, high frequency of the p.R326Q mutation in Japanese patients, and relatively high prevalence of the p.R326Q mutation in the Japanese population, suggests there may be additional undiagnosed patients with βUP deficiency. Thus, pyrimidine degradation defects should be included in differential diagnosis of unexplained neurological abnormalities, such as convulsions, developmental delay, autism and related disorders.

## Electronic supplementary material

Below is the link to the electronic supplementary material.

ESM 1 (DOCX 17 kb)
