# Supplementary material for: Clinical, biochemical and molecular analysis of 13 Japanese patients with β-ureidopropionase deficiency demonstrates high prevalence of the c.977G > A (p.R326Q) mutation
Source: J Inherit Metab Dis. 2014 Feb 14;37(5):801–12. doi: 10.1007/s10545-014-9682-y (PMC4158181; doi:10.1007/s10545-014-9682-y)
Supplement: Supplementary file 1 — (DOCX 17 kb) [file 10545_2014_9682_MOESM1_ESM.docx]

**Table 1S.** Primers used for site-directed mutagenesis of *UPB1*

| Mutation | Primer name | Primer sequences |
| --- | --- | --- |
| G31S | g91a  g91a -antisense | 5’-gtgaagcgcgttctctat**a**gcaaggaactcaggaagc-3’  5’-gcttcctgagttccttgc**t**atagagaacgcgcttcac-3’ |
| E271K | g811a  g811a-antisense | 5’-gtccctgtggcccatc**a**aggccagaaacgcagc-3’  5’-gctgcgtttctggcct**t**gatgggccacagggac-3’ |
| I286T | t857c  t857c-antisense | 5’-gcttcacctgcgcca**c**caatcgagtgggcac-3’  5’-gtgcccactcgattg**g**tggcgcaggtgaagc-3’ |
| R326Q | g977a  g977a-antisense | 5’-cccctgacagcagcc**a**gactcctgggctgtc-3’  5’-gacagcccaggagtc**t**ggctgctgtcagggg-3’ |
